# Activation of TRPV2 and BKCa channels by the LL-37 enantiomers stimulates calcium entry and migration of cancer cells

**DOI:** 10.18632/oncotarget.8122

**Published:** 2016-03-16

**Authors:** Audrey Gambade, Sami Zreika, Maxime Guéguinou, Igor Chourpa, Gaëlle Fromont, Ana Maria Bouchet, Julien Burlaud-Gaillard, Marie Potier-Cartereau, Sébastien Roger, Vincent Aucagne, Stéphan Chevalier, Christophe Vandier, Caroline Goupille, Günther Weber

**Affiliations:** ^1^ Inserm, UMR1069, Nutrition, Croissance et Cancer, Tours, France; ^2^ Department of Medical Lab Technology, Jinan University, Tripoli, Lebanon; ^3^ Ion channel network Canceropole Grand Ouest; ^4^ Université François Rabelais, Tours, France; ^5^ CHRU Hôpital Bretonneau, Tours, France; ^6^ Centre de Biophysique Moléculaire, CNRS UPR 4301, Orléans, France

**Keywords:** LL-37, calcium signaling, membrane association, cell migration, breast cancer

## Abstract

Expression of the antimicrobial peptide hCAP18/LL-37 is associated to malignancy in various cancer forms, stimulating cell migration and metastasis. We report that LL-37 induces migration of three cancer cell lines by activating the TRPV2 calcium-permeable channel and recruiting it to pseudopodia through activation of the PI3K/AKT pathway. Ca^2+^ entry through TRPV2 cooperated with a K^+^ efflux through the BKCa channel. In a panel of human breast tumors, the expression of TRPV2 and LL-37 was found to be positively correlated. The D-enantiomer of LL-37 showed identical effects as the L-peptide, suggesting that no binding to a specific receptor was involved. LL-37 attached to caveolae and pseudopodia membranes and decreased membrane fluidity, suggesting that a modification of the physical properties of the lipid membrane bilayer was the underlying mechanism of its effects.

## INTRODUCTION

The peptide LL-37 is released from the C-terminus of the Human Cathelicidin Antimicrobial Protein hCAP18. Apart from its antimicrobial activities, it also induces proliferation and migration of epithelial cells or stimulates angiogenesis by a direct effect on endothelial cells (for review, [1]).

In multiple cancer forms [2, 3] LL-37 was found to stimulate both proliferation and migration of cancer cells, thus contributing to cancer development and progression. LL-37 was proposed to promote cell migration through stimulation of FPRL-1 receptor, a Pertussis toxin-sensitive G protein coupled receptor (GPCR) in cell lines from ovarian cancer and human skin [4]. In pancreatic cancer cell lines the ATP-gated purinergic receptor P2X7 was additionally required to induce cell invasion [5]. In several other cell lines LL-37 activated different tyrosine kinase receptors and ERK1/2 and AKT signaling [6–8]. In the weakly invasive MCF7 breast cancer line, LL-37 increased cell mobility and metastasis development in a mouse model without involving FPRL-1 [3]. Although the activation of MAPK signaling via ERBB2, and/or IGFR [9] was observed in this model system, the mechanism underlying this induction was not identified. Taken together, LL-37 has the ability to activate multiple plasma membrane receptors of unrelated structures, and to induce a pro-metastatic phenotype.

Remodeling of Ca^2+^ homeostasis is an important modifier of migratory and invasive activities in cancer cells [10–12]. In non-excitable cells, Ca^2+^ entry mostly occurs through non-voltage-gated Ca^2+^ channels located in the plasma membrane. Some Ca^2+^-permeable channels can be activated after binding their ligand, such as the ATP-gated P2X receptors [13]. Store-Operated Ca^2+^ Channels (SOCs) are activated by preliminary release of internal Ca^2+^ stores that induce Store-Operated Ca^2+^Entry (SOCE). In comparison, Store-Independent Ca^2+^ Channels (SICs) can be stimulated without the release of internal Ca^2+^ stores. These mechanisms are supposed to mainly activate the channels of the TRP superfamily and the Orai family [14]. Some of these channels are constitutively open at the plasma membrane contributing to basal migration and invasivity in breast and prostate cancer cells [14]. Ca^2+^ entry can activate Ca^2+^-activated K^+^ channels leading to membrane hyperpolarization, thus increasing the driving force for Ca^2+^ across the plasma membrane [15]. To date, two K^+^ channels (hEag1 and SK3) that potentiate Orai1-dependent constitutive Ca^2+^ entry have been reported to stimulate cancer cell migration [16] and metastasis [17].

LL-37 induces Ca^2+^ mobilization in mast cells [18] and in submandibular gland cells [19]. The only Ca^2+^ channel identified so far as activated by LL-37 is the P2X7 receptor channel. In a transgene HEK293 model system [20], Ca^2+^-influx through P2X7 increased cell proliferation.

The goal of this study was therefore to investigate whether the effects of LL-37 on cancer cell migration might be linked to regulation of intracellular Ca^2+^ concentration, to identify plasma membrane Ca^2+^ channels activated by LL-37 and potentially cooperating K^+^ channels, and to determine their mechanism of activation by LL-37.

## RESULTS

### LL-37 binds to the plasma membrane and induces Ca^2+^ entry and cell migration

**Figure 1:**
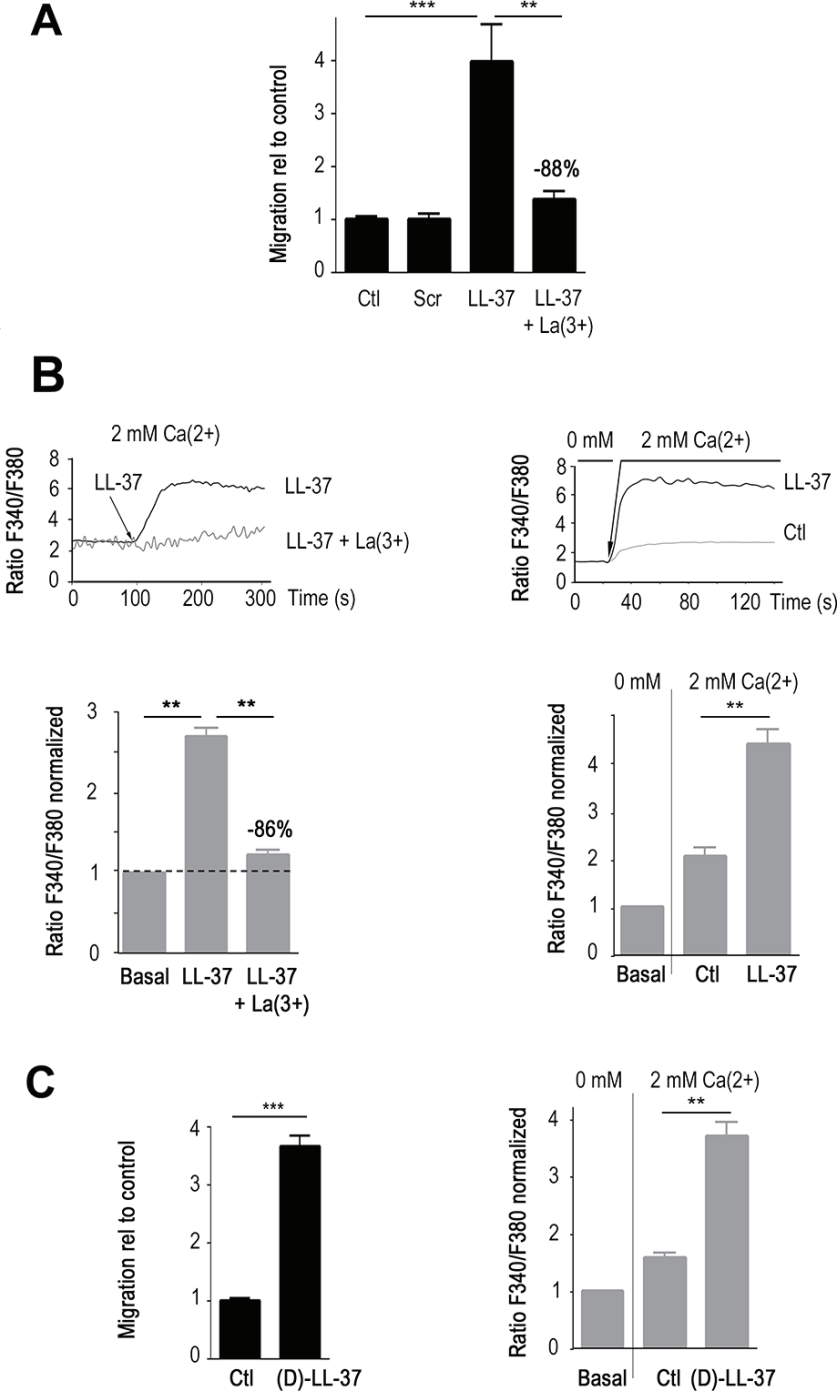
LL-37 induces Ca^2+^ influx that promotes migration of MDA-MB-435s cells (**A**) LL-37 induced migration is blocked by La^3+^. The level of induction by LL-37 in presence and absence of 100 μM LaCl_3_ (La), and scrambled peptide (scr) is displayed relative to cell migration (Ctl) without LL-37. *n* ≥ 8. (**B**) LL-37 increases intracellular Ca^2+^. Upper graphs showing the time course of fura-2 fluorescence ratio detected at 510 nm with both excitations at 340 and 380 nm. Lower graphs, compilations of experiments where the fluorescence ratio is normalized against the basal level of each experiment. Left panels display results obtained using a constant external 2 mM Ca^2+^. The dotted line shows the normalized basal level and La^3+^ inhibitory effect is indicated relative to LL-37 alone (*n* ≥ 6). Right panels display results obtained by shifting the extracellular Ca^2+^ from 0 to 2 mM after 20s of measurement without depletion of the intracellular store. Ctl : fluorescence ratio without LL-37 after applying of 2 mM Ca^2+^, LL-37 : fluorescence ratio with LL-37 after applying of 2 mM Ca^2+^. *n* = 10. (**C**) (D)-LL-37 induces cell migration (left panel, *n* = 10). By shifting the extracellular Ca^2+^ from 0 to 2 mM, (D)-LL-37 increases fura-2 fluorescence ratio detected at 510 nm with excitations at 340 and 380 nm.(right panel, *n* = 7).

We initially focused on the highly malignant cancer cell line MDA-MB-435s, for which several Ca^2+^-permeant channels had already been characterized as mediators of cell migration [17]. In accordance with our previous findings in MCF7 cells [3], LL-37 induced cell migration of MDA-MB-435s at 10 μg/ml (≈ 4 fold increase, *p* < 0.001, Figure 1A). A scrambled peptide with identical content in amino acids showed no effect. Lanthanum (La^3+^), a non-specific blocker of Ca^2+^ channels, significantly reduced the cell migration by 88% (*p* < 0.01).

As shown by Ca^2+^ spectrofluorimetry in presence of 2 mM external Ca^2+^ (Figure 1B, left panels), intracellular Ca^2+^ increased after few seconds upon treatment with LL-37. This was significantly decreased by incubation with La^3+^ (−86%, *p* < 0.01, Figure 1B, left panel). A modification of the protocol [17] permitted us to measure the constitutive entry of extracellular Ca^2+^ through active Ca^2+^channels of the plasma membrane, without depletion of the intracellular Ca^2+^ stores. Compared to control condition, which showed a Ca^2+^ influx through constitutively open channels, LL-37 substantially increased this Ca^2+^ entry (Figure 1B, right panel).

A peptide with opposite chirality ((D)-LL37) presented identical activities in increasing internal Ca^2+^ and migration of MDA-MB-435s (Figure 1C). These results suggested that specific peptide-protein interactions were not required and that the cellular effects of LL-37 might originate from its capacity to attach to the membrane.

**Figure 2:**
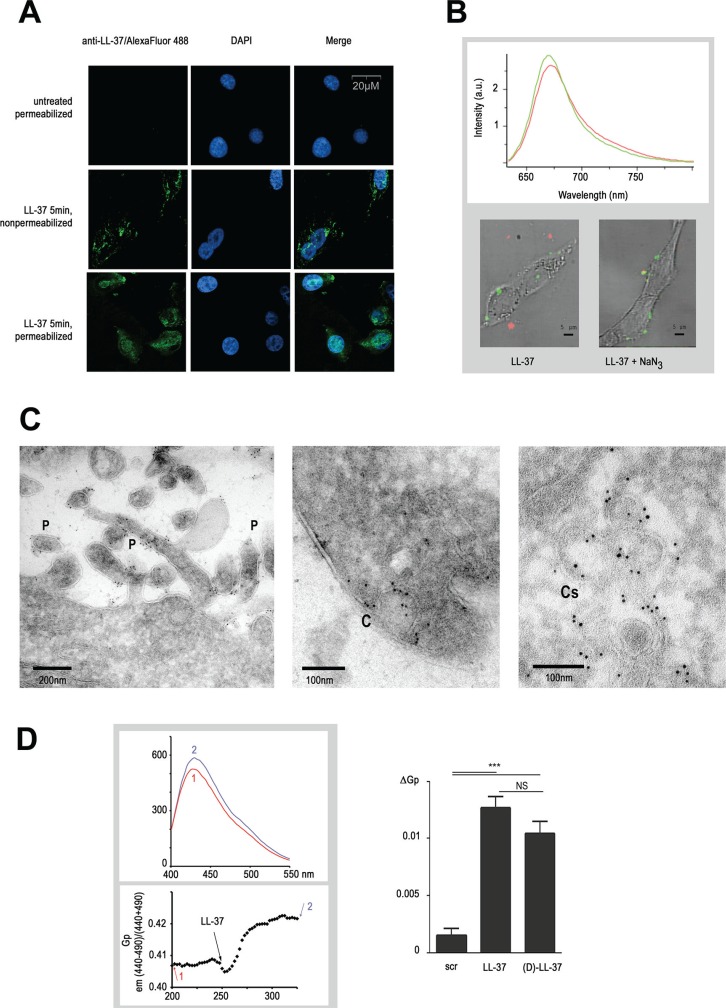
LL-37 binds to pseudopodia and caveolae membranes altering their fluidity (**A**) LL-37 attachment to plasma membrane and its internalisation after 5 min. Detection of LL-37 by immunofluorescence performed on non-permeabilized and permeabilized MDA-MB-435s cells treated with LL-37 for 5 min. DAPI is used for nuclear staining. (**B**) Localisation of LL-37 in an apolar environment at and inside the MDA-MB-435s cells. Labelling of LL-37 using azido-functionalized LL-37 (LL-37-Asp26Ile) coupled with Cy5 fluorochrome (Cy5fluo-LL-37) detected by confocal microspectrometry. The graph shows the characteristic emission (Cy5fluo-LL-37), measured in a apolar (green) environment when attached to the cell or a polar (red) environment when located outside. Images below show the superposition of a visible cell image with its Cy5fluo-LL37 spectral card after 5 min, in absence (left) or presence (right) of 1% NaN_3_. (**C**) Localisation of LL-37 on pseudopodia and caveolae membranes in the MDA-MB-435s cell by immunoelectron microscopy. Cells were incubated with LL-37 for 5 min, fixed and then immunogold labelling was performed. Identification of pseudopodia (left, structures indicated with a P) and caveolae (mid, indication with (C) at the extracellular membrane, and of caveosome membranes at the intracellular membrane (right, indicated with Cs). (**D**) Increase of the generalized polarisation value of by LL-37. The upper graph shows an emission spectrum of the Laurdan probe before (1) and after (2) treatment of MDA-MB-435s cells with LL-37. The lower graph shows the time course of GP measurement as described in materials and methods, arrows with numbers (1) and (2) indicating when the emission spectrum (upper graph) was recorded. Right diagram shows the compilation of ΔGP value after addition of scrambled (scr), (L)-LL-37 or (D)-LL-37 peptides (*n* ≥ 8).

These findings prompted us to determine where LL-37 bound to the cell. The initial immunofluorimetric analysis revealed that LL-37 attached to the plasma membrane (Figure 2A, LL-37 on non-permeabilized cells), but was partially endocytosed by the cell after 5 min of incubation (Figure 2A, LL-37 on permeabilized cells). To follow the fate of LL-37 in the living cell, the Cy5 fluorochrome was conjugated to a peptide modified at position 26 by an azido-functionalized amino acid. Using a bioorthogonal strain-promoted azide/alkyne cycloaddition reaction, conjugation could be conducted either before or after application of the peptide to the cells. Both alternatives resulted in identical localization of the peptide in the cell (data not shown). We had previously verified that an amino acid exchange at this position (LL-37 Asp26Ile) did not change its effect (data not shown) on cell migration and activation of Ca^2+^ influx. Confocal microspectral analysis revealed a significant blue shift by 3 nm on the emission spectrum when Cy5fluo-LL-37 was localized on the surface of the cell (green spectrum and green zones on Figure 2B), compared to the same peptide in culture medium (red spectrum and red zones, Figure 2B). This spectral shift is characteristic for a decreased polarity environment of the fluorochrome [21]. This suggested that LL-37 bound to the plasma membrane, and remained located to a membrane after intracellular uptake of the peptide. Sodium azide (1%), a potent inhibitor of ATP-driven endocytosis, did not prevent Cy5fluo-LL-37 from binding to the cell but blocked its internalization (Figure 2B, bottom right).

Immunogold-labeled anti-LL-37 was located at the surface of pseudopodia and invaginated structures characteristic of caveolae as assayed in immunoelectron microscopy (Figure 2C, left and middle panels), thus confirming the association of LL-37 to membrane structures. Apart from these structures, no signal was found on the remaining extracellular membrane. Intracellular signals in cells treated with LL-37 for 5 min remained exclusively at membranes of the caveosomes (Figure 2C, right panel) without any evidence of free cytoplasmic LL-37.

The activity of membrane-associated proteins and signal transduction is influenced by the organization of the plasma membrane, signaling being more active in rigid nanodomains (such as lipid rafts) than more fluid phases [22, 23]. We investigated the effect of LL-37 on the plasma membrane fluidity with Laurdan, a lipid-packaging sensor [24, 25]. The generalized polarization (GP) value, which permits a quantitative assessment of the membrane order, increased by 2.5% (from 0.4 to 0.41) after addition of LL-37, indicating a decrease of membrane fluidity (Figure 2D). In comparison, lysophosphatidylcholine at 100 μM, known to increase membrane fluidity [26], reduced the GP value by 10% (data not shown).

To assess the effect of LL-37 on membranes of different compositions and phase states [27], the GP values of large unilamellar vesicles were determined after addition of increasing concentrations of LL-37. LL-37 decreased membrane fluidity for all conditions assayed (Supplementary Figure S1).

### LL-37 recruits TRPV2 to pseudopodia through the PI3K pathway

The Orai1/SK3 channel complex has been characterized as a source for a constitutive Ca^2+^ entry in MDA-MB-435s cells [17]. Orai1 suppression by RNA interference reduced the constitutive Ca^2+^ entry, however at a similar rate during absence or presence of LL-37 (Supplementary Figure S2A). Orai1 thus appeared active irrespective of the presence of LL-37, and thus not responsible for the additional Ca^2+^ entry caused by LL-37. When expressed in HEK293 cells, P2X7 has been reported to act as a Ca^2+^ permeant channel activated by LL-37 [20]. P2X7 is fully functional in MDA-MB435s cells, in which it promotes cell migration and invasiveness [28, 29]. However, KN62, an antagonist of P2X7, did not prevent the Ca^2+^ entry induced by LL-37 (data not shown).

**Figure 3:**
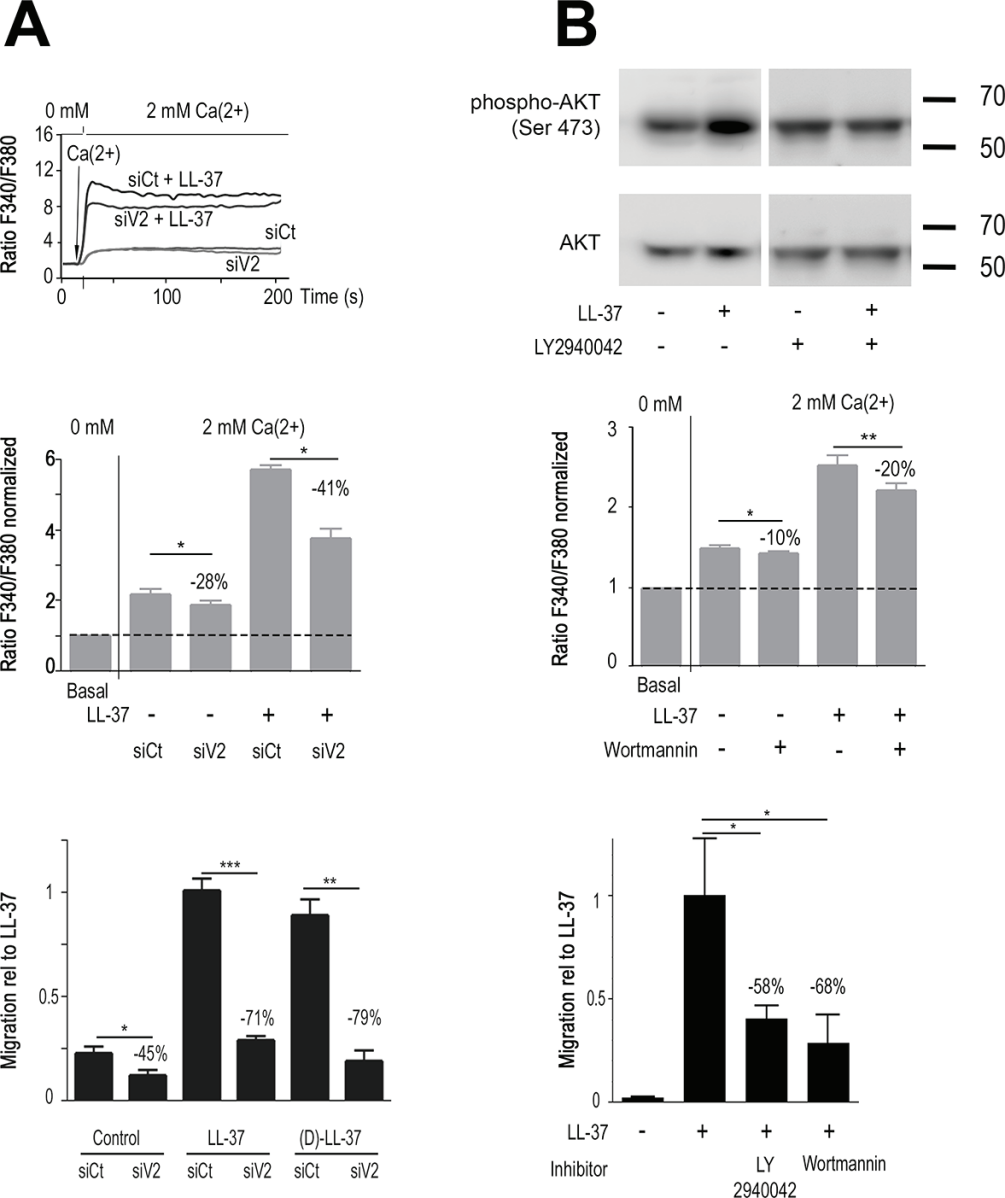
LL-37 increases PI3K/AKT signaling and Ca^2+^-influx through TRPV2, promoting MDA-MB-435s cell migration (**A**) RNA interference against TRPV2 reduces Ca^2+^ entry and cell migration caused by LL-37. Upper panel: time course of intracellular Ca^2+^ by shifting the extracellular Ca^2+^ from 0 to 2 mM after 20s on cells treated with siRNA against TRPV2 (siV2) or control siRNA (siCt) in presence and absence of LL-37. The mid panel displays compilation of results. Fura-2 fluorescence ratio is normalized against the basal level of each individual experiment. The dotted line shows the normalized basal level and the inhibitory effect of siV2 is indicated relative to siCtl with LL-37 (*n* = 8). Bottom panel: (L-)LL-37- and (D)-LL-37 induced (*n* ≥ 8) cell migration is suppressed by TRPV2 siRNA. Values are displayed relative to the migration of cells induced by LL-37. FCS is used to control cell mobility in presence of siRNAs (*n* = 3). (**B**) PI3K/AKT pathway activation by LL-37 contributes to Ca^2+^ entry and cell migration. Upper panel: Western blot analysis of AKT phosphorylation in MDA-MB-435s protein extracts treated or not with LL-37 and/or PI3K inhibitor LY2940042 (1 μM). Blots were reprobed with a panAKT antibody. Mid panel: Ca^2+^ entry by LL-37 is decreased in presence of PI3K inhibitor Wortmannin (*n* ≥ 10). Condition and evaluation as above. Bottom panel: Suppression of LL-37 induced cell migration in presence of 1 μM LY294002 (*n* = 8) or 100 nM Wortmannin (*n* = 5). Values are displayed relative to the migration of cells induced by LL-37.

In order to identify other putative targets for LL-37, we screened the expression of Ca^2+^ channels previously identified as involved in various cancer cell properties [11]. The TRPV2 and TRPC1 channels showed a notably high expression (Supplementary Figure S3). Suppression of TRPC1 by RNA interference had no effect on LL-37 induced Ca^2+^ entry (Supplementary Figure S2B). In contrast, the suppression of TRPV2 by RNA interference resulted in significant reduction of LL-37 induced cell migration and Ca^2+^ entry (Figure 3A). At basal conditions, siTRPV2 reduced constitutive Ca^2+^ entry by 27%, whereas the induction by LL-37 was reduced by 41% (*p* < 0.01, Figure 3A, upper and middle panels).

In cells treated with siTRPV2, basal migration without peptide was reduced (−45%) but clearly at less extent than LL-37-stimulated migration. The pro-migratory effects of (D)- and (L)-peptides were both suppressed at similar extent (−71% and −79% respectively, *p* < 0.001, Figure 3A, lower panel), confirming their identical mechanism of activity.

As TRPV2 has been reported activated by PI3K/AKT signaling [30, 31] we investigated whether LL-37 could induce this pathway. Western blot analysis revealed that AKT phosphorylation was induced by LL-37, and blocked by the PI3K inhibitor LY2940042 (Figure 3B, upper panel). In agreement with this, the PI3K inhibitor Wortmannin partially inhibited LL-37 induced Ca^2+^ entry (−20% *p* < 0.01, Figure 3B, middle panel). Both inhibitors, Wortmannin and LY2940042, strongly reduced LL-37 induced cell migration (−68% and −58%, respectively, *p* < 0.01, Figure 3B, lower panel).

Previous experiments in MCF7 cells had demonstrated that LL-37 could activate ERK/MAPK signaling [3]. In MDA-MB-435s, ERK was found constitutively phosphorylated, and the ERK inhibitor UO126 did not block LL-37-stimulated migration (data not shown).

**Figure 4:**
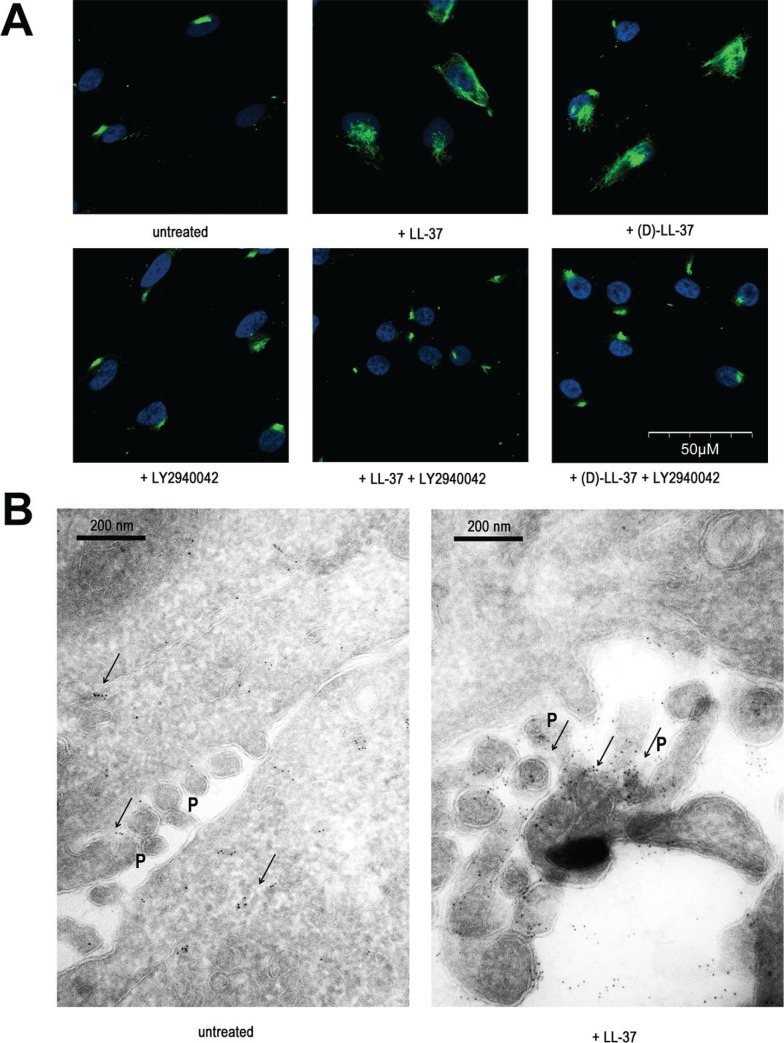
LL-37 induces PI3K-dependent TRPV2 translocation to pseudopodia membranes (**A**) Immunofluorescence analysis on non-permeabilized cells revealing the translocation of TRPV2 to the plasma membrane by (L)-LL-37 and (D)-LL-37 peptides and blocked by PI3K inhibitor LY2940042 (1 μM). (**B**) Localisation of TRPV2 by immunoelectron microscopy. The location of immunogold signals, intracellularly and sporadically at pseudopodia (P) before and accumulated at pseudopodia after 5 min of cell treatment with LL-37, is indicated with arrows.

TRPV2 has been shown to be translocated to the plasma membrane upon activation [31], or being localized to the membrane and providing constitutive Ca^2+^ entry [32]. We studied the location of TRPV2 by fluorescence microscopy on immunostained MDA-MB-435s cells. In permeabilized unstimulated cells, TRPV2 was located predominantly intracellularly (data not shown), but a weak spotted staining was observable on the membrane surface at non-permeabilized cells (Figure 4A). This localization is in accordance with the constitutive Ca^2+^ entry that was suppressed by RNA interference (Figure 3A). Upon a 5-min long LL-37 treatment TRPV2 labeling strongly increased at the plasma membrane, suggesting it was translocated from intracellular compartments. Inhibitors of AKT/PI3K (LY2940042 or Wortmannin) prevented this translocation to the plasma membrane. Identical results were obtained when (D)-LL-37 was used instead of its natural (L)-enantiomer (Figure 4A).

We performed immunoelectron microscopy to precise TRPV2 cellular localization. In unstimulated MDA-MB-435s cells, TRPV2 staining was predominantly intracellular, in proximity of the plasma membrane and associated with membrane structures including Golgi vesicles. Some spots were also observed in pseudopodia. Upon LL-37 treatment, the staining at pseudopodia was dramatically increased, at the expense of the intracellular compartment (Figure 4B). Taken together, these results are consistent with a membrane translocation of TRPV2 to specialized structures such as pseudopodia upon LL-37 exposure.

### The BKCa channel cooperates with TRPV2

**Figure 5:**
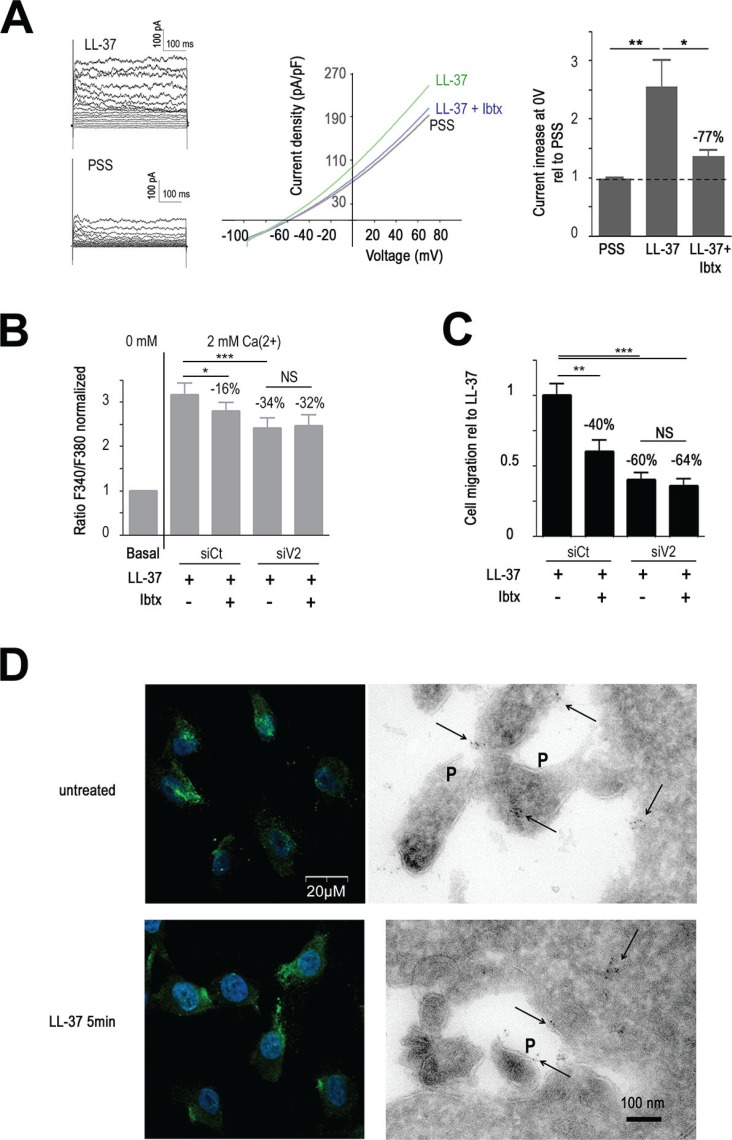
TRPV2 cooperates with BKCa (**A**) BKCa inhibitor IbTx decreases LL-37 induced outward potassium current on MDA-MB-435s cells. Left panel shows examples of currents obtained with I-V protocol on MDA-MB-435s cells in presence (LL-37) or absence (PSS) of the peptide. Currents were measured for each voltage clamp (V) from −90 at +80 mV during 500 ms, every 10 mV increase. Mid panel displays a representative Current-Voltage curves which shown the current density-voltage relationships obtained using ramp protocol recorded in PSS or in presence of LL-37 and with or without pretreatment with 100 nM Ibtx. Right panel represents the compilation of the outward current at the membrane voltage at 0 mV normalized to PSS condition (5 < *n* < 12). (**B**) IbTx decreases LL-37 induced Ca2+ influx without additional effect of anti-TRPV2 siRNA (siV2). Fura-2 probe is used to measure Ca^2+^ influx after 2 mM Ca^2+^ application as described in Materials and Methods. The effects of LL-37 and IbTx (100 nM) are evaluated on MDA-MB-435s cells transfected with siRNA against TRPV2 or control siRNA (siCt). Fura-2 fluorescence ratio is normalized against the basal level of each individual experiment. The dotted line shows the normalized basal level and the inhibitory effect of siV2 and/or IbTx is indicated relative to siCtl with LL-37 (*n* = 13). (**C**) IbTx decreases LL-37 induced cell migration without additional effect of anti-TRPV2 siRNA (siV2). The inhibitory effect of siV2 and/or IbTx is indicated relative to siCtl with LL-37 (*n* = 8). (**D**) BKCa membrane location remains unaltered after application of LL-37. Left panels show the detection of BKCa by immunofluorescence performed on non-permeabilized MDA-MB-435s cells treated or not with LL-37 for 5 min. DAPI is used for nuclear staining. Right panels show the localisation of BKCa on pseudopodia (P) and intracellularly by immunoelectron microscopy. The location of immunogold signals, intracellularly and at pseudopodia (P) is indicated with arrows.

Since the driving force of Ca^2+^ entry can be promoted by K+ efflux [33], we performed an initial study using a tetraethylammonium (TEA) as non-selective inhibitor of K^+^ channels. TEA reduced the intracellular Ca^2+^ increase induced by LL-37 (Supplementary Figure S2C) suggesting a contribution of K^+^ channels. Previous experiments by Chantôme et al. [17] have shown that constitutive Ca^2+^ entry in MDA-MB-435s cells is supported by the SK3 channel which was removed by transformation with a lentivector expressing short hairpin RNA (shRNA) against SK3. In this shRNA-SK3 cell model, the Ca^2+^ entry induced by LL-37 was weakly decreased, suggesting that this channel was not deeply activated by LL-37 (Supplementary Figure S2D). We therefore investigated the large conductance Ca^2+^ activated K^+^ channel (BKCa), which is also expressed in these cells [34]. Figure 5A is a typical example of current-density voltage relationships showing an increase of the amplitude of an outward current upon LL-37 treatment. This outward current, measured at the membrane voltage of 0 mV (Figure 5A, right panel), was increased by LL-37, reaching a 2.5 fold induction. This induction was significantly reduced (−77%) by pretreatment with iberiotoxin (Ibtx), suggesting BKCa as responsible for the current.

In correspondence, Ibtx reduced both Ca^2+^ entry (−16%, Figure 5B) and cell-migration (−40%, Figure 5C) induced by LL-37. The constitutive Ca^2+^ entry remained unchanged by Ibtx. Interestingly, when combining BKCa inhibition by Ibtx and the suppression of TRPV2 by RNA interference, no further reduction of the activities by LL-37 was obtained. This suggested that the activities of both channels were linked to each other and not on unrelated pathways. Confocal microscopy located BKCa at the plasma membrane, and this remained apparently unaltered by treatment with LL-37 (Figure 5D, left panel). Using electronic microscopy BKCa immunogold signals appeared intracellular and on the membranes of pseudopodia (Figure 5D, right panel).

### LL-37 and TRPV2 cooperate in breast tumors and cell cancer lines

**Figure 6:**
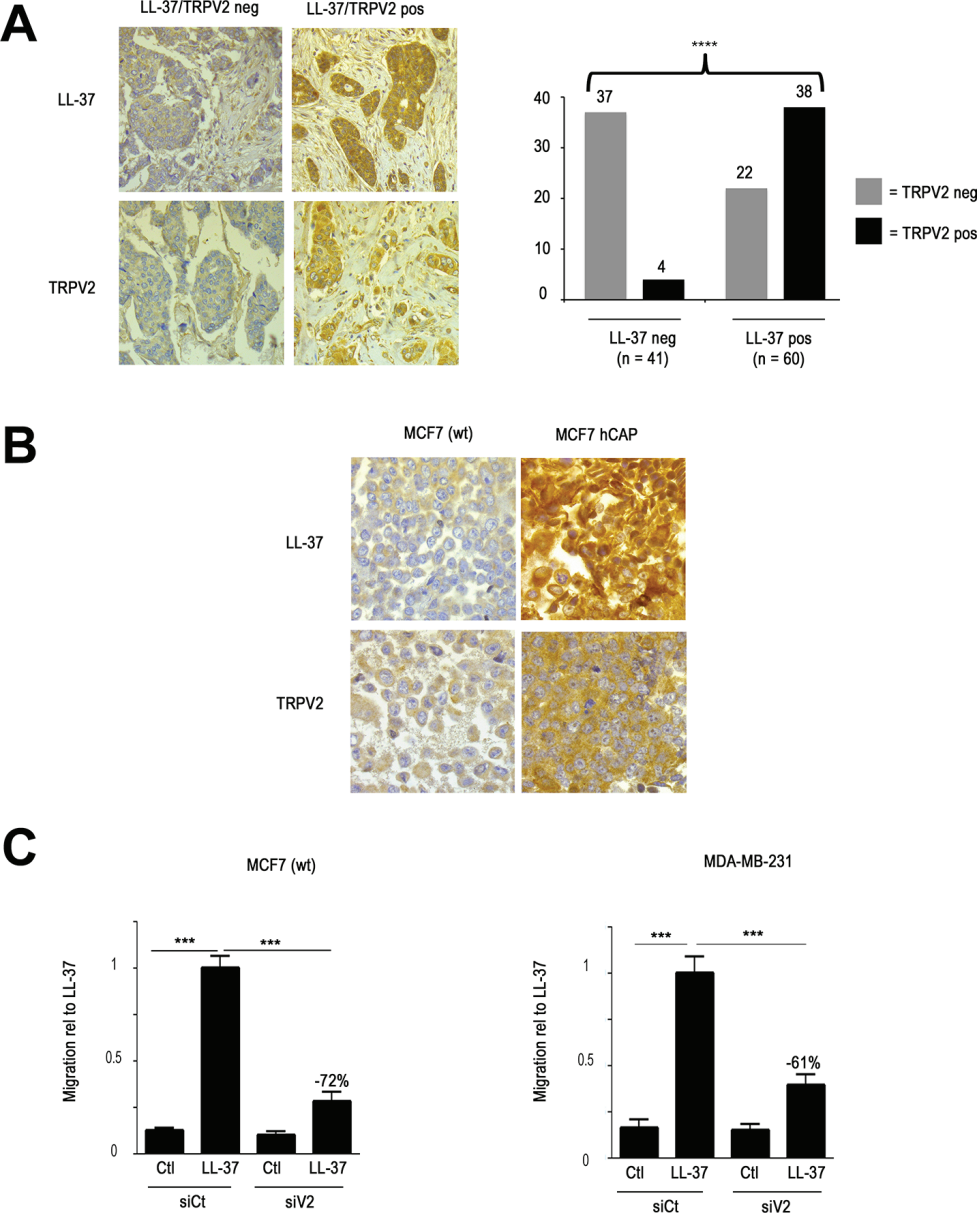
LL-37 and TRPV2 cooperate in cell cancer lines and breast tumors (**A**) Immunohistochemical analysis of breast tumors reveals coexpreussion of LL-37 and TRPV2. Left panel: representative example of sections from two breast carcinomas showing strong signals for both LL-37 and TRPV2 staining, or low immunoreactivity for either of them. Right panel displays the evaluation of 101 breast tumors, and revealing significant coexpression of LL-37 and TRPV2 (chi-square: *p* < 10^−4^). (**B**) Immunohistochemistry on the MCF7 cell line, revealing an increased signal for TRPV2 in cells with transgenic expression of hCAP18/LL-37. (**C**) RNA interference against TRPV2 decreases LL-37 induced cell migration of MCF7 (left panel) and MDA-MB-231 (right panel) cells.

To investigate whether TRPV2 and LL-37 may be connected in breast cancer, we performed an immunohistochemical study on sections of 101 breast tumors (Figure 6A). Staining for LL37 and TRPV2 was found in 60 and 42 invasive carcinomas, respectively. The signal of both was significantly correlated (*P* < 10^−4^). In the group of LL-37-positive tumors, the number of tumors positively staining for TRPV2 was clearly enriched. An even more striking correlation was found in LL-37 negative tumors, of which only 4 of 41 tumors expressed TRPV2 in absence of LL-37.

Based on such a tight correlation, we hypothesized that the expression of LL-37 might directly influence the level of TRPV2. Since MDA-MB-435s cells highly expressed TRPV2, we studied the MCF7 cell line, which showed low basal expression of TRPV2. We created a transgenic derivative by stable transfection with an expression vector for hCAP18/LL-37. Immunohistochemistry revealed an increased signal for TRPV2 as well (Figure 6B). Further analysis by qRT-PCR did not show any altered transcription level, indicating that the influence of LL-37 on TRPV2 levels was posttranscriptional (data not shown).

In order to confirm whether the relation between LL-37 and TRPV2 held true on the functional level as well, we performed a migration study on MCF7, and in addition on the high malignant MDA-MB-231 breast cancer cell line. LL-37 induced migration in both cell lines (Figure 6C) as well as the (D)-enantiomer (data not shown). In both cases this induction was suppressed by RNA interference against TRPV2 (Figure 6C).

## DISCUSSION

**Figure 7:**
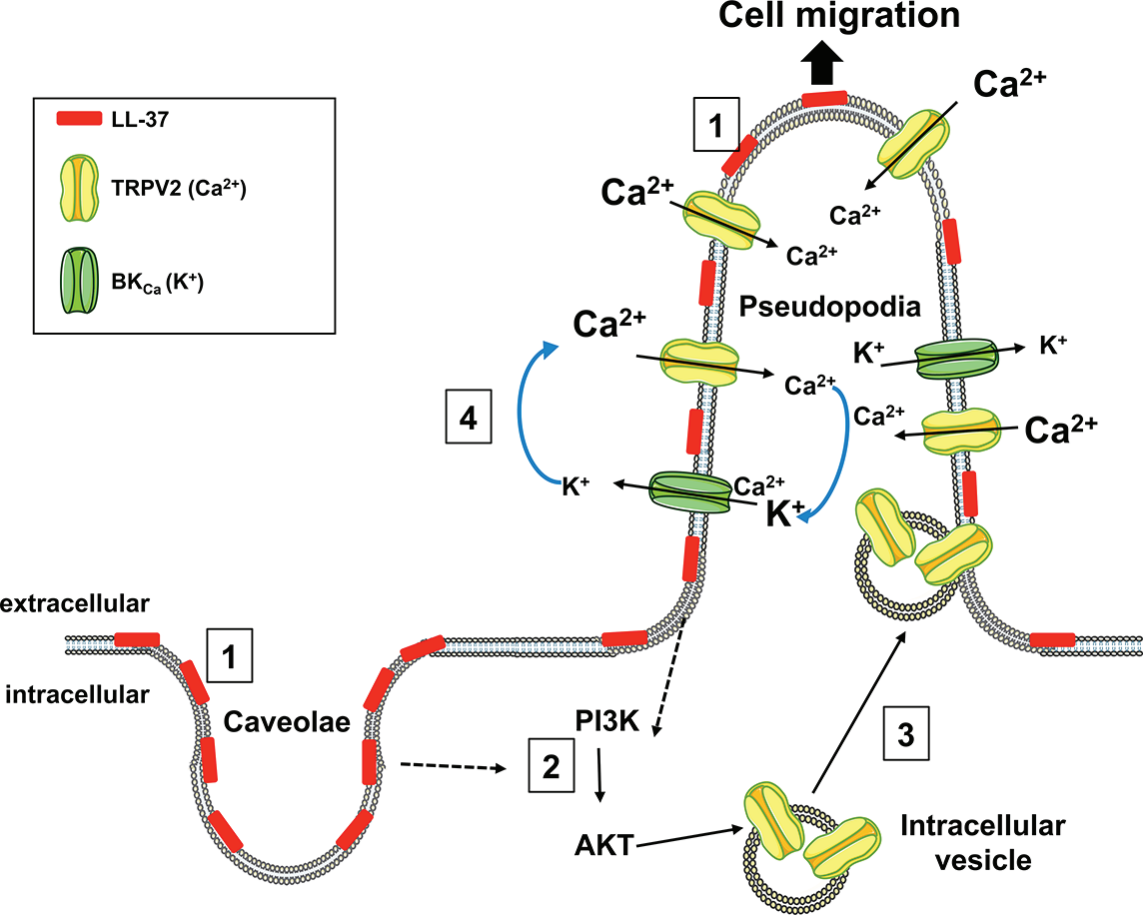
Mechanism proposed for the stimulatory activity of LL-37 on cell migration LL-37 binds to the membranes of caveolae and pseudopodia (**1**), and activates PI3K/AKT signaling (**2**). AKT induces the recruitment of the TRPV2 channel (**3**) from intracellular vesicles to plasma membranes of pseudopodia. The increase of intracellular Ca^2+^ induced by TRPV2 is accompanied by K^+^ efflux through BKCa (**4**), which preserves the ion balance and helps to maintain the Ca^2+^ entry, which promotes cancer cell migration.

We have shown that LL-37 binds to the plasma membrane, especially in caveolae and pseudopodia. This leads to increase intracellular Ca^2+^ and cell migration through activation of the Ca^2+^ channel TRPV2 predominantly located in pseudopodia. Moreover, LL-37 induces TRPV2 recruitment at the plasma membrane by a PI3K/AKT-dependent pathway. Figure 7 summarizes our data with the activated channels involved for migration, their cellular location as well as the mechanisms of action we propose for LL-37.

In accordance with results of two previous studies [6, 20], both (D)- and (L)- enantiomers presented identical activities. As mentioned above, previous studies have shown a variety of receptors being activated by LL-37, however without detailing evidence for a conventional receptor-ligand interaction. It needs to be considered whether some of these observations can equally be explained by an indirect activation of the respective receptors through interaction of LL-37 with the cell membrane.

Both increase and decrease of membrane fluidity can activate signaling pathways on the same cell [35]. Fluidity alterations in either direction have been observed in cancer, depending of the cancer type and stage of carcinogenesis and no general trend being discerned [36]. Epithelial-to-mesenchymal transition and cell migration of breast cancer lines are reported to be suppressed by globally decreasing membrane fluidity [37, 38]. On the migrating cell, however, membrane rigidity was shown locally increased at leading edge and retracting tail [39]. The latter observation is in line with our findings, especially when considering that LL-37 acts locally on pseudopodia, structures related to cell migration.

We have localized LL-37 at the cellular membrane specifically of caveolae and pseudopodia, and remaining at the caveosome after its internalization. While this manuscript was being revised, the binding of LL-37 in complex with lipopolysaccharides and their endocytosis at lipid rafts was published to require sulfated proteoglycans [40] on the cell surface, which may have served to guide the attachement of cationic LL-37 attachment.

The peptide forms an amphipathic helix, attaching on the membrane surface in form of a carpet [41, 42]. LL-37 binding can induce a mechanical stress on the plasma membrane [43]. This may explain how TRPV2, known to be an osmo- [44] and mechanosensor [45, 46] channel, is activated. Mechanical stress has been shown to locally increase PI3K activity at the sites of the stress, required to recruit TRPV2 to the membrane [46], and to activate the channel [32]. We found LL-37 attached to both caveolae and pseudopodia, whereas TRPV2 was exclusively directed to the pseudopodia. The binding of LL-37 to the caveolae and its modification of the membrane structure may activate the receptors localized therein [39], and lead to AKT signaling that addresses the channel to pseudopodia. Whether the localization of LL-37 to caveolae and/or pseudopodia directs the translocation of TRPV2 can presently not be determined.

It should be mentioned that part of TRPV2 was already located in pseudopodia in basal conditions, which explains its contribution to the constitutive Ca^2+^ entry and migration without stimulation by LL-37.

The inhibition of the BKCa channel decreased Ca^2+^ entry and cell migration, however, without an additive effect when both BKCa and TRPV2 were simultaneously suppressed. We suggest that Ca^2+^ entry through TRPV2 activates the Ca^2+^-sensitive BKCa channel increasing the driving force of Ca^2+^ across TRPV2. We have presently no evidence about a formation of a physical complex between TRPV2 and BKCa channels but both were found in pseudopodia, supporting their functional association.

Although we cannot exclude a participation of other Ca^2+^ channels, TRPV2 appears to be the major player for LL-37 induced migration in the three cell lines we have investigated. Our immunohistochemical studies on breast tumors show a significant correlation of LL-37 and TRPV2 expression indicating a physiological relevance of their cooperation. ‘Transgenic expression of hCAP18/LL-37 in MCF7 cells increased TRPV2 by a posttranscriptional mechanism, which may explain why both were found coexpressed in breast tumors.

Expression of TRP channels has been correlated with the aggressivity of multiple cancer forms, both in model systems [47] and in clinical investigations [48, 49]. Similarly LL-37 has been linked to development and progression of various cancer forms. Our results have shown a functional association between LL-37 and ion channels, which appears as a novel approach for research on cancer development and drafting of therapeutic strategies.

## MATERIALS AND METHODS

### Cell lines

MCF7, MDA-MB-435s and MDA-MB-231 cell lines were obtained from ATCC and grown in Dulbecco's modified Eagle's medium, supplemented with 5% fetal calf serum (Eurobio, Courtaboeuf, France). Cells were grown at 37°C in a humidity saturated atmosphere containing 5% CO_2_.

For transgenic expression of hCAP18, MCF7 cells were transfected with a pIRES-EGFP expression vector [2] and selected with G418 at 400 μg/ml and by FACS sorting.

### Peptides, inhibitors and antibodies used in this study

Unmodified L- and D-peptides were synthesized and HPLC-purified to > 95% (GeneCust, Dudelange, Luxembourg, and GL Biochemicals, Shanghai, China). Sequences were: LLGDFFRKSKEKIGKEFKRIVQRIK DFLRNLVPRTES for LL-37, and RSLEGTDRFPFVRL KNSRKLEFKDIKGIKREQFVKIL for the scrambled control peptide [50]. LL-37-Asp26Ile (GL Biochemicals) was used to verify that modifications at position 26 did not affect the activity of this peptide. All experiments were performed at 10 μg/ml (2.2 μM) of peptides. Inhibitors and antibodies and the concentration used in this study are listed in Supplementary Tables S3 and S4.

### Cell migration assay

Before cell migration assay, cells were starved 24 h in DMEM BSA 0.1% and FCS 0.1% and were seeded (15 000 cells for MDA-MD-435, 15 000 for MDA-MB-231, 50 000 for MCF7) in the upper side of a migration chamber (Insert with Polyethylene filter with 8 μm pores, Falcon BD Biosciences, Le Pont de Claix, France). The lower chamber was filled with DMEM BSA 0.1% and FCS 0.1% added or not with LL-37 (10 μg/ml). After 4 h at 37°C for MDA-MB-231 and MDA-MB-435s cells, cells having migrated in the lower face of the filter were fixed with methanol, labelled with hematoxyline (Sigma-Aldrich, St. Quentin Fallavier, France) and counted. Given the lower migratory capacity of MCF7 cells, their migration was performed over 14 hours. To highlight the pro-migratory effect of LL-37, results are normalized to migration of cells without LL-37. To highlight potential inhibitory effects of reagent or siRNA or inhibitors, results are normalized to migration of cells with LL-37.

### Intracellular Ca^2+^ measurements

Cells were seeded at 600 000 cells in 28 cm^²^ tissue culture dishes 48 h before the experiment and kept in OptiMEM (Life Technologies, Saint Aubin, France) containing 0.2% BSA during 24 h. Cells were loaded with the ratiometric dye Fura2-AM (5 μM) (Thermo Scientific, Illkirch, France) at 37°C for 45 min, then detached with EDTA and centrifuged. Two protocols were available to follow intracellular Ca^2+^ concentration: for the first, cells were re-suspended in physiological saline solution (PSS) (NaCl 140 mM, KCl 4 mM, MgCl_2_ 1 mM, HEPES 10 mM and Glucose 11.1 mM, pH 7.4) containing 2 mM Ca^2+^. This leads to measure total intracellular Ca^2+^ concentration variations. Fluorescence emissions of cell suspensions in a magnetically stirred cuvette were monitored in a spectrophotometer (F-2710 FL, Hitachi/VWR, Fontenay-sous-Bois, France). Pharmacological inhibitors (concentrations in Supplementary Table S4) were added at the beginning of the measurement, and after a stabilization period of 300 s (used as Ca^2+^ intracellular basal level), LL-37 was injected. Intracellular Ca^2+^ variations were evaluated using the fluorescence (510 nm) emission ratio at excitations at 340 and 380 nm. The basal ratio obtained for Ca^2+^ concentration was used as reference to normalize subsequent measurements after LL-37 application with or without inhibitors.

For the second protocol, which selectively monitors the import of extracellular Ca^2+^ across the plasma membrane, cells were suspended in PSS without Ca^2+^. LL-37 was added at the beginning of the measurement (used as basal level) and after 20 s 2 mM Ca^2+^ was applied. Intracellular Ca^2+^ concentration variations were detected as described above. In this protocol, inhibitors were incubated during loading with Fura2-AM, and additionally added at resuspension of the cells before the beginning of the measurement.

### Determination of cell membrane fluidity changes

Cells were kept in Optimem overnight, mechanically detached by flushing with DPBS (Lonza, Levallois-Perret, France). A suspension of 500 000 cells/ml in DPBS was incubated for 15 min at 37°C with 5 μM Laurdan (Sigma-Aldrich), and fluorescence emissions were determined in the spectrophotometer as above, at an excitation at 365 nm. The Generalized Polarisation (GP) value was determined as: GP = (em_440_−em_490_)/(em_440_+em_490_), em being the emission intensities at the indicated wavelengths [25].

### Immunofluorescence labeling

Cells were seeded in Lab-Tek chamber 8-well slides at 20 000 cells per well during 48 h. 24 h before treatment, cells were kept in OptiMEM, BSA 0.2%. Cells were washed with DPBS with Ca^2+^ and incubated with LL-37 10 μg/ml at room temperature. After 5 min, LL-37 was removed, cells were fixed with DPBS containing 4% paraformaldehyde during 15 min at 4°C under agitation, and incubated with saturation solution (DPBS containing 50 mM NH_4_Cl and 3% BSA) or permeabilisation solution (DPBS, 50 mM NH_4_ Cl, 3% BSA 0.1% Tween 20) 30 min at 4°C. Anti-LL-37 rabbit antibody (Osenses/Ozyme, Fontenay-sous-Bois, France) was diluted in DPBS, 1% BSA at 1:500 and added on cells overnight at 4°C under agitation. Next, cells were incubated with secondary antibody coupled with green fluorescence dye CF488A (Biotium/Ozyme) at 1:2000 for 2 h. After staining with DAPI (Ref 32670, Sigma-Aldrich) at 1/5000 for 2 min, samples were washed with DPBS and lamella were fixed with Fluorescent Mounting Medium (DAKO, Les Ulis, France).

For the TRPV2 labeling protocol, cells were pre-incubated or not with inhibitors during 45 min at 37°C. LL-37 or (D)-LL-37 were added during 5 min at room temperature. Anti TRPV2 rabbit antibodies (extracellular domain, Antibodies Online, Aachen, Germany) at 1:300 and secondary antibodies coupled with CF488A at 1/200 were used for labelling TRPV2 as described above. Cells were observed on a confocal microscope, at ×600 magnification (Olympus Fluoview FV500 Laser Scanning Confocal Biological Microscope) and image acquisition was performed using Fluoview 500 v.5 software (Olympus, Tokyo, Japan).

### *In vitro* and *in cellulo* fluorescence labeling of an azido-functionalized LL-37

A modified peptide (LL-37-Asp26Anl) at which Asp26 had replaced by ω-azidonorleucine, was synthesized on a Prelude automated synthesizer (Protein technologies) starting from an aminomethyl PEGA resin (Novabiochem/Merck Millipore, Molsheim, France) and using standard Fmoc/*t*-butyl chemistry. *N*-Fmoc-ω-azido-L-norleucine was prepared as described [51]. Standard trifluoroacetic acid-activated cleavage of the resin and removal of the side chains protecting groups was followed by purification of the peptide to > 99% purity by reverse phase HPLC. For fluorescence labelling, DBCO-Sulfo-Cy5 (Jena Bioscience, Jena, Germany) was coupled to LL-37-Asp26Anl through a bioorthogonal strain-promoted azide/alkyne cycloaddition (SPAAC) reaction according to the manufacturer's protocols, either before or after application of the peptide to the cells at 10 μg/ml. Coupling of the fluorochrome before or after treatment of the cells with functionalized LL-37 did not to influence its cellular localization (data not shown).

### Confocal spectral imaging

25000 cells were seeded on cover slides placed in 24-well plates for overnight. The medium was discarded and the cells were incubated for 30 min with Cy5-labelled modified LL-37 (10 μg/ml in DPBS) at 37°C and 5% CO_2_. After incubation, cells were washed thrice with fresh DPBS, put on a microscope glass slide, covered with a drop of DPBS and with a thin cover slide.

Fluorescence measurements were carried out using a low dispersion mode (grating 300 grooves/mm) of a laser-scanning LabRam confocal microspectrometer (Horiba, Les Ulis, France). Cy5 fluorescence was excited with the 632.8 nm line of a built-in air-cooled, He-Ne laser. The power on the samples was estimated to be below 150 μW; the acquisition time was 0.02 s per spectrum. No sample photodegradation was observed. Sample irradiation and collection of fluorescence spectra were performed through a × 50 LWD microscope objective (numerical aperture 0.90; Olympus). The confocal whole aperture was adjusted to obtain ~0.8 μm lateral and 3.5 μm axial resolution.

For each cell analysis, an optical section (x–y plane) situated at half-thickness of the cell was scanned with a step of 0.8 μm and at each point a full fluorescence spectrum from 635 to 800 nm was recorded. That provided maps containing typically 625 spectra (25 × 25 points). Both acquisition and treatment of multispectral maps were performed with LabSpec software. Subcellular LL-37 distribution maps were established via analysis of both the intensity and shape of Cy5 fluorescence spectra, as described [52]. Briefly, each experimental spectrum was fitted using the least-squares method to a sum of the two reference spectra of Cy5 where the shorter/longer position (maxima at 668/671 nm) corresponded respectively to a lower/higher polarity of the molecular environment of the fluorophore [21]. The fitting errors were below 5% (typically 2–4%). These coefficients were used to generate the respective two-dimensional distribution maps over the cell. The results were averaged over 9–12 cells for each kind of treatment. The cellular autofluorescence was completely neglected, because of the absence of any significant fluorescence of the untreated cells under the conditions used.

### Immunogold labelling of cryosections according to Tokuyasu for immunoelectron microscopy [53]

Cells were cultured in 175 cm^2^ flask at 80% confluence during 48 h. 24 h before treatment, cells were starved with OptiMEM containing 0.2% BSA. Cells were suspended in DPBS with Ca^2+^ and treated with LL-37 or not treated. After 5 min, PFA at 8% is added directly on suspended cells v/v to final concentration at 4% and cells were incubated 2 hours at room temperature stirring then washed in DPBS for 2 × 5 min and centrifuged at 2000 × g for 10 min. After removing the supernatant, cell pellets were included in gelatin 12% and infused with sucrose 2.3 M overnight at 4°C. 90 nm ultra-thin cryosections were made a −110°C on a LEICA UCT cryoultramicrotome. Sections were retrieved with Methylcellulose 2%/Sucrose 2.3 M mixture (1:1) and collected onto formvar/carbon coated nickel grids. After removal of gelatine at 37°C, sections were incubated on drops of DPBS, 1% BSA and one hour on drops of DDPBS with 1:200 antibody anti-LL-37 (Osenses) or 1:100 antibody anti-TRPV2 (Antibodies Online). After six washes of five minutes each, grids were incubated on drops of DPBS containing 1:30 gold-conjugated (6 nm) goat-anti-rabbit IgG (Aurion/Biovalley, Nanterre, France). Grids were finally washed with six drops of DPBS (five minutes each), post-fixed in 1% glutaraldehyde and rinsed with three drops of distilled water. Contrasting step was performed by incubating grids on drops of uranyl acetate 4%/methylcellulose 2% mixture (1:10). The sections were imaged in a transmission electron microscope at 100 kV (JEOL 1011).

### Immunohistochemistry

For immunohistochemistry of cell lines,15 million cells were harvested, spun down, dehydrated and embedded in paraffin. Slide sections from cells and tumor samples were deparaffinized, rehydrated in 10 mM sodium citrate pH 6.0, and treated in a microwave oven (600 W) for 3 × 10 min. After treatment with 3% of H_2_O_2_ for 5 min, slides were blocked in TBS/10% FCS for 30 min, then primary antibody was applied in TBS/0.1% TritonX100 at 4°C. Antibodies and concentrations are listed in Supplementary Table S3 The specificity of the TRPV2 antibody was validated on MDA-MB-435s cells with/without suppression of TRPV2 by RNA interference. Treatment with secondary antibody and development was performed with a commercial detection kit (Dako).

For immunohistochemistry on tumors, the study cohort included 101 female patients which consent was obtained prior to the study. The age median age was 49 years (28 minimum and 71 maximum). They were treated at the university Hospital of Tours (France) and presented breast cancer proven by immunohistopathology. A pathologist with experience in breast disorders reviewed histology and staining on slides. The histological type of the cancers was all invasive with 82 ductal carcinomas (81, 2%), 7 lobular carcinomas (6.9%) and 12 carcinomas with other histotypes (11.9%) (such as cribriform, tubular, mucinous…). 81 carcinomas expressed oestrogen and/or progesterone receptors (80,2%), 10 were classified as HER positive (9.9%) and 16 as triple negative phenotype (15.8%). TRPV2 and LL-37 labelling was performed as specified for cell lines (see above).

### RNA interference

siRNAs used in this study are listed in Supplementary Table S1. For TRPC1 and TRPV2, two additional control siRNAs were used against different target sites of the transcripts in initial experiments on intracellular Ca^2+^ and on cell migration, to verify the specificity of our observations. The silencing efficiency was monitored by qRT-PCR or Western Blot analysis and exceeded 70% for all siRNA. A scrambled siRNA (Qiagen) served in all experiments as a transfection control.

Cell lines were transfected in suspension using Lipofectamine RNAiMax (Fisher Scientific) according to the manufacturer's protocol, at a final siRNA concentration of 30 nM. Cells were then seeded in tissue culture dishes 28 cm² at 750 000 cells per dish or 6-wells plates at 250 000 cells per well (in culture medium). Experiments were performed 48 h after transfection.

### Expression analysis by quantitative real-time polymerase chain reaction (qRT-PCR)

Total RNA of MDA-MB-435s, MDA-MB-231 or MFC-7 cells were extracted according to standard protocols with NucleoSpin^®^RNA II kit (Macherey-Nagel, Hoerdt, France), and reverse transcribed using RevertAid First Strand cDNA Synthesis Kit (Fermentas/Thermo Scientific). Gene quantifications were performed on 50 ng of cDNA using SYBR^®^ Premix Ex Taq^™^ II (Tli RNaseH Plus, TAKARA/Ozyme) using MyiQ thermocycler (Bio-Rad, Marnes-la-Coquette France). Primers (all from Sigma-Aldrich) were used at final concentration of 200 nM and temperature protocol was 10min start at 95°C, followed by 40 cycles of 15 s at 95°C, 30 s at 57°C and 30 s at 72°C. Relative gene expression levels were normalized to HPRT1 and calculated using the 2^−DDCt^ method. Primers are listed in Supplementary Table S3.

### Electrophysiological recordings

MDA-MB-435s cells were seeded at 10 000 cells per 35 mm Petri dish 48 h before the experiment and serum-starved in OptiMEM supplemented with 0.2% BSA during 24 h. Before measurement, extracellular culture medium was replaced by physiological saline solution PSS (140 mM NaCl, 4 mM KCl, 1 mM MgCl_2_, 2 mM CaCl_2_, 0.33 mM NaH_2_PO_4_, 10 mM HEPES and 11.1 mM glucose, pH 7,4). Experiments were performed at room temperature using conventional whole-cell configuration as previously described [17]. Briefly, Patch-clamp experiments were performed with an Axopatch 200B patch-clamp amplifier (Axon Instruments, Wokingham, UK). Data are recorded with 1322-A Digidata converter (Axon Instruments) and pClamp software (v9.2, Axon Instruments) was used for generation of voltage commands, acquisition and analysis of whole-cell currents. Patch pipettes were pulled from borosilicate glass capillary (3–5 MΩ) by a stretcher (P-97 model, Sutter Instrumens, Cancale, France) and were filled with a intrapipette solution at pCa 6.4 (125 mM K-Glutamate, 20 mM KCl, 1 mM MgCl_2_, 1 mM Mg-ATP, 0.7 mM CaCl_2_, 1 mM EGTA and 10 mM HEPES, pH 7.2). During experiments, the cell was continuously perfused with PSS or PSS containing LL-37 or/and inhibitors.

MDA-MB-435s whole-cell currents were measured using two different protocols. First, a current-voltage (I–V) protocol was performed and the membrane was clamped by steps from −90 at +80 mV during 500 ms with 10 mV increments. We next used a ramp protocol from −100 to +70 mV during 500 ms, from a holding potential of 0 mV and 4 s between each ramp. Currents (I) were normalized to cell capacitance and expressed as densities of current (pA/pF). The conductance was estimated as a slope of the I–V curves around the inversed potential of cells. Current amplitudes were analyzed at 0 mV. The patch-clamp data were then analyzed using OriginPro software (OriginLab, Paris, France).

### Western blot analysis

Cells were rinsed in ice-cold DPBS before protein extraction with Sodium dodecylsulfate lysis buffer containing 1% β-mercaptoethanol, and heated 5 min at 72°C. Western blot was essentially performed as described [3]. Samples were separated using electrophoresis gel at 8% poly-acrylamide and transferred to nitrocellulose membrane (GE Healthcare), and incubated with primary antibody solutions overnight at 4°C. Antibodies and their dilutions are listed in Supplementary Table S3. After incubation with HRP-coupled secondary antibodies and application of ECL advance solution (GE Healthcare/Fisher Scientific) signals were recorded with a CCD camera (MF ChemiBIS, DNR Bio-imaging Systems, Jerusalem, Israel) and evaluated using Multi Gauge Software (v3.0, Fujifilm, Tokyo, Japan). For normalisation, filters were stripped with 200 mM glycine/HCl pH 2.5, and reprobed.

### Statistic analysis

The experiments were evaluated by Mann-Whitney and paired Wilcoxon statistics. Statistical significance is indicated in the figures with stars, *p < 0.05, **p < 0.01, ***p < 0.001. The number n of measurements for the individual experiments is indicated in the figure legends. Results are displayed as mean ± SEM. The Chi-square test was used for comparison between immunohistochemical staining of LL-37 or TRPV2 and the characteristics of the 101 carcinomas.

## SUPPLEMENTARY MATERIALS FIGURES AND TABLES


