# Supplementary material for: Activation of TRPV2 and BKCa channels by the LL-37 enantiomers stimulates calcium entry and migration of cancer cells
Source: Oncotarget. 2016 Mar 16;7(17):23785–800. doi: 10.18632/oncotarget.8122 (PMC5029663; doi:10.18632/oncotarget.8122)
Supplement: Supplementary file 1 [file oncotarget-07-23785-s001.pdf]

Activation of TRPV2 and BKCa channels by the LL-37 enantiomers stimulates calcium entry and migration of cancer cells

Supplementary Materials

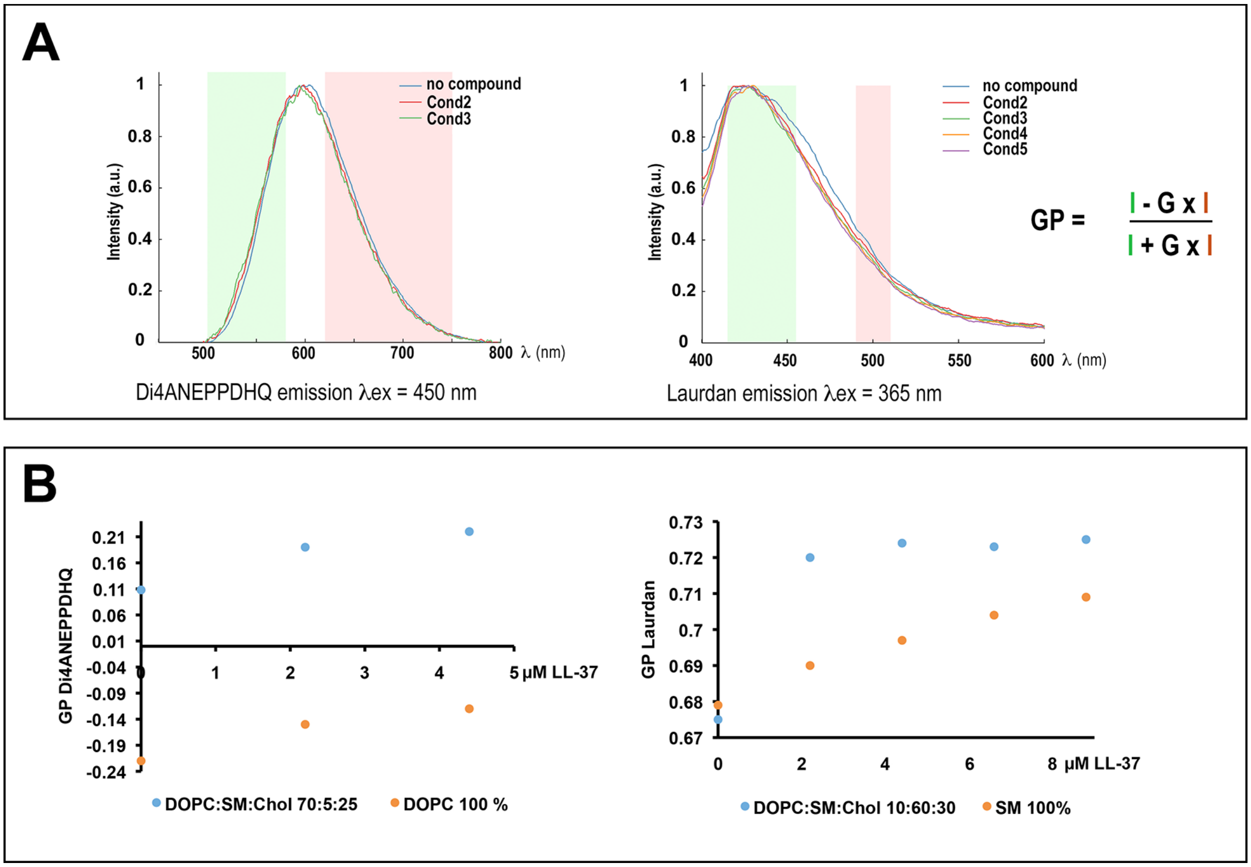

Supplementary Figure S1: LL37 increases the GP values in Large Unilamellar Vesicles in all lipid phase states. Large Unilamellar Vesicles of 100 nm diameter were prepared at a 150 µM concentration with different % lipid molar ratio of: 1, 2-Dioleoyl-sn-glycero-3-phosphocholine (DOPC), cholesterol (Chol), and egg sphingomyelin (SM, containing ≈86% N-palmitoyl SM) in order to get liquid ordered/gel (DOPC:SM:Chol 10:60:30/SM 100%) or liquid disordered/fluid (DOPC:SM:Chol 70:5:25/ DOPC 100%) phase state. All compositions were incubated with Laurdan or Di4ANEPPDHQ at a Lipid/Probe = 300 molar ratio. The changes in the *GP* value were calculated in all conditions after addition of increasing concentrations of LL37 using the equation reported in graph (**A**) and shown in graph (**B**) The average for each condition is shown for both probes with a SEM ± 0.01.

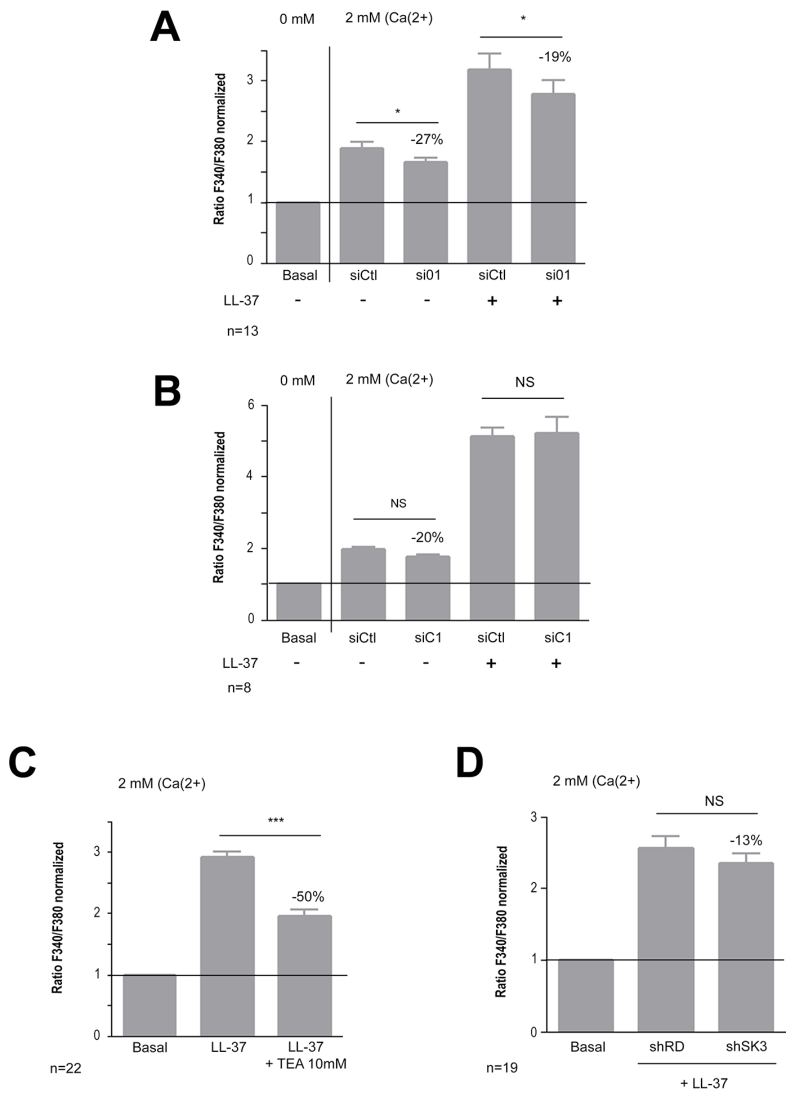

Supplementary Figure S2: LL-37 increases intracellular Ca2+ independent of Orai1 and TRPC1 channels but dependent of K+ channels in MDA-MB-435s cells. (**A**) When shifting the extracellular Ca2+ from 0 to 2 mM after 20 s of measurement without depletion of the intracellular store, siRNA against Orai1 (siO1) decreases constitutive Ca2+ entry and LL-37-induced Ca2+ influx in the same manner. Fura-2 fluorescent ratio is normalized against the basal level of each experiment. The line shows the normalized basal level and the inhibitory effect of siO1 is indicated relative to control siRNA (siCtl) in the same condition. (**B**) siRNA against TRPC1 (siC1) decreases constitutive Ca2+ entry but has no effect on LL-37-induced Ca2+ influx. Evaluation performed as above. (**C**) LL-37-induced Ca2+ influx is decreased when K+ channels are inhibited by TEA using a constant external 2 mM Ca2+. The dotted line shows the normalized basal level and TEA inhibitory effect is indicated relative to LL-37.

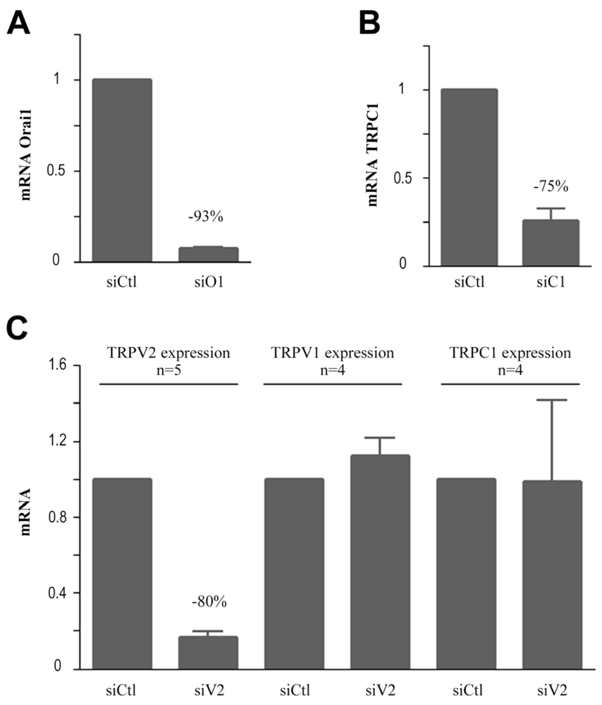

Supplementary Figure S3: Efficiency of siRNA on expression of Orai1, TRPC1 and TRPV2 as measured by qRT-PCR of transfected MDA-MB-435s cells. (**A**) siRNA against Orai1 (siO1) decreases mRNA expression level of Orai1 by 93%. (**B**) siRNA against TRPC1 (siC1) decreases mRNA expression level of TRPC1 by 75%. (**C**) siRNA against TRPV2 (siV2) decreases mRNA expression level of TRPV2 by 80% but has no effect on mRNA expression levels of TRPV1 and TRPC1. mRNA expression levels are evaluated by RT-qPCR as described in Materials and Methods.

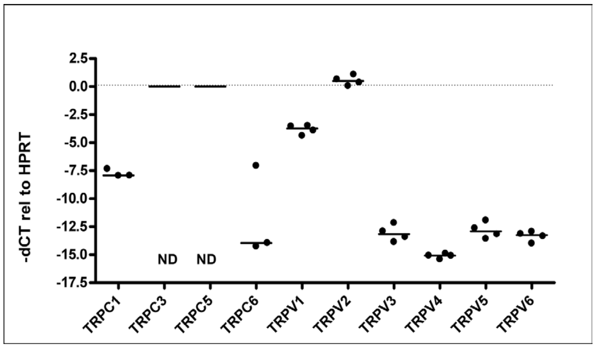

Supplementary Figure S4: mRNA expression of TRPC/TRPV family members in MDA-MB-435s cells as determined by qRT-PCR. Values are displayed as relative (-dCt) to the HPRT housekeeping gene.

Supplementary Table S1: List of siRNAs used in this study

Target gene	Target sequence (nt)	Manufactorer/provider
TRPC1	5′-CATGGAGCATCATATTTCA-3′	Eurogentec, Angers, France
TRPC1 controls	5′-GACTGACACAACTGTATGA-3′<br>5′-CTTTCGGACTTCTAAATAT-3′	
TRPC6	5′-CGTTCTTTATGGAGTCTAT-3′	
TRPC6 control	5′-GCAATGAACTGGCAGTTCT-3′	
TRPV2	5′-GAACCTGCTTTACTATACA-3′	
TRPV2 controls	5′-GTGATGATCTCGGACAACT-3′<br>5′-CACGTGTTCATCTGGATCT-3′	
Orai1		Santa Cruz Biotechnologies Heidelberg, Germany,<br>cat c-76001
scrambled		Qiagen, Courtaboeuf , France<br>cat 1027281

Supplementary Table S2: List of primers used for qRT-PCR

Gene	Forward primer 5′-3′	Reverse primer 5′-3′
TRPC1	TGGGATGATTTGGTCAGACA	TCTGCCACCAGTGTAGGATG
TRPC3	CTTTCCTTCAGGCAACGAAG	GTACGCAATCCGAGAGAAGC
TRPC5	CAACTGTCGTGGAATGGATG	CCCTTGGACGAGAACCATTA
TRPC6	GGATTACATGGGCCAGAATG	GCTGGTTGCTAACCTCTTGC
TRPV1	CAACAAGATCGCACAGGAG	TCCTTGCCATCAGGTGTGTA
TRPV2	GGAATACACAGAGGGCTCCA	CCTCTTCTCAATGGCGATGT
TRPV3	ACGAGGCAACAACATCCTTC	CCGCTTCTCCTTGATCTCAG
TRPV4	CCCGTGAGAACACCAAGTTT	AGTTCATTGATGGGCTCCAC
TRPV5	GGAGCTTGTGGTCTCCTCTG	GAAACTTAAGGGGGCGGTA
TRPV6	TCAAGCCCAGGACCAATAAC	GTCCAAAGAAGCGAGTGACC
ORAI1	CTGATCATGAGCGCAAACAG	ATGGTGGCAATGGTGGAG
HPRT1	TGACCTTGATTTATTTTGCATACC	CGAGCAAGACGTTCAGTCCT

Supplementary Table S3: List of antibodies and conditions of use in this study

Primary antibodies		
**Target protein, position, species**	**Manufactorer/provider**	**Method and dilution**
TRPV2, 3rd extracellular loop, rabbit	antibodies-online.com,<br>cat ABIN351263	Immunofluorescence, 1/300 electron microscopy: 1/100<br>Immunohistochemistry: 1/50
BKCa, extracellular, rabbit	Alomone Laboratories, Jerusalem, Israel,<br>cat APC 107	Immunofluorescence: 1/200<br>electron microscopy 1/100
LL-37 whole peptide, rabbit	Osenses, Keswick, Australia,<br>cat. OSC00030P	Immunofluorescence: 1/500 electron microscopy: 1/200
phosphoAKT (Ser473), rabbit	Cell signaling/Ozyme, cat 4060	Western blot: 1/1000
panAKT, mouse	Cell signaling/Ozyme, cat 2920	Western blot: 1/1500
**Secondary antibodies**		
CF488A chicken anti-rabbit	Biotium, cat 20209	Immunofluorescence: 1/2000
Gold-conjugated (6 nm) goat anti-rabbit	Aurion, cat 806.011	electron microscopy: 1/30
Goat anti-rabbit IgG-HRP	Santa Cruz, cat sc-2004	Western blot: 1/4000
Goat anti-mouse IgG-HRP	Santa Cruz, cat sc-2005	Western blot: 1/4000

Supplementary Table S4: List of inhibitors used in this study

Inhibitor	Manufactorer/provider	concentration
Iberiotoxin (Ibtx)	Santa Cruz, Cat sc-3585	50 nM
Lanthanum(III) chloride heptahydrate ($LaCl_3$)	Sigma-Aldrich, cat 262072	100 µM
Sodium azide (NaN_3)	Sigma-Aldrich, cat S8302	1%
KN62	Calbiochem/Merck-Millipore, cat 422706	100 nM
LY-2940042 hydrochloride	Sigma Aldrich, cat L9908	1 µM
Wortmannin	Calbiochem/Merck-Millipore, cat 681676	100 nM
UO126	Calbiochem/Merck-Millipore	1 µM
Tetraethylammonium chloride (TEA)	Sigma-Aldrich, cat T2265	10 mM
